# Effect of probiotics and paraprobiotics on patients with sleep disorders and sub-healthy sleep conditions: a meta-analysis of randomized controlled trials

**DOI:** 10.3389/fneur.2024.1477533

**Published:** 2024-10-16

**Authors:** Bei Yu, Ke-Yi Wang, Ning-Rui Wang, Lu Zhang, Jian-Ping Zhang

**Affiliations:** ^1^The Third School of Clinical Medicine, Zhejiang Chinese Medical University, Hangzhou, China; ^2^Department of Anatomy, Histology and Embryology, School of Basic Medical Sciences, Zhejiang Chinese Medical University, Hangzhou, China

**Keywords:** probiotics, paraprobiotics, sleep disorders, adults, meta-analysis

## Abstract

**Background:**

The microbial-gut-brain axis has received much attention in recent years, and regulating intestinal flora can effectively improve sleep disorders, which hints the potential effects of probiotics on sleep disorders, but lack of research evidence for meta-analysis. Therefore, this study aims to quantitatively evaluate the influence of probiotics on sleep disorders and sub-healthy sleep conditions.

**Methods:**

Up to 2023, online databases including Pubmed, Embase, Cochrane library, Web of science have been searched for studies involving adults who consume probiotics or paraprobiotics in controlled trials, during which, changes in subjective and/or objective sleep parameters and contributing factors in sleeping quality are examined. We conduct a meta-analysis of 11 clinical randomized controlled studies.

**Results:**

Probiotic supplementation improves sleep states to some extent in adults with sleep disorders and healthy adults with condition-induced sleep disorders (−0.34 [−0.56 to −0.13]; *I*^2^ = 42.6%; *p* = 0.001). Meanwhile, subgroup analysis shows that the effect of probiotics on improving sleep disorders is influenced by other factors such as the health states of the subjects, the duration of the intervention, the type of strain, and the test criteria.

**Conclusion:**

Probiotics and paraprobiotics have a significant positive effect on the sleep quality of adults with sleep disorders or sub-healthy sleep conditions. However, the therapeutic effects of probiotics on sleep problems need future additional trials.

**Systematic review registration:**

https://inplasy.com/inplasy-2022-12-0066/, identifier 2022120066.

## 1 Introduction

Sleep disorders are divided into eight categories and the four major sleep complaints include excessive daytime sleepiness, insomnia, abnormal movements or behavior during sleep and inability to sleep at the desired time (1). The problems of sleep disorders are very common in people of all ages and have gradually become a global public health epidemic (2). According to reports, approximately 29% of adults in the United States of America are reported to sleep <7 h per night (3), 50–70 million people have chronic sleep and wakefulness disorders, and about 83.6 million adults sleep less than 7 h within 24 h (4). A survey about population sleep health from developed countries indicates that more than one-third of adults are regularly sleep deprived (5–7). Otherwise, sleep disorders can lead to various unhealthy consequences, such as cardiovascular disease, declining of cognition, and a serious of mental disorders like anxiety and depression and so on (8). A study from Poland (9) shows that the sleep quality in patients with temporomandibular disorders is affected by pain, which significantly reduces life satisfaction. Consequently, it’s evident that sleep quality is intimately linked to life quality. In addition, in adults, sleep disturbances can lead to a range of poor health outcomes, including increased risk for falls and all-cause mortality (10). Sleep disorders not only exist in adults, but also widely exist in children, which has a great impact on children’s growth and development. A study (11) has pointed out that children’s sleep behavior problems are related to oral manifestations such as temporomandibular disorders, if persisted for a long time, there will be adverse effects on children’s stomatognathic system. The increasing number of people with sleep disorders can also cause related social and economic pressures (12). However, it’s limited to completely reverse the symptoms by current drug treatments. Therefore, it’s also urgent to find an effective therapy for sleep disorders.

Study (13) has shown that regulating and optimizing the structure of the gut microbiota can improve sleep disorders. Another study (14) on insomnia has indicated that adjusting the composition of the gut microbiota can improve sleep disturbances, with the mechanism “gut-brain axis.” The interaction between the gut microbiota and the central nervous system (CNS) exists in the so-called “gut-brain axis” (15), and the gut microbiome participates in sleep physiology through producing an extensive array of metabolites and other compounds which have neuroactive and immunomodulatory properties such as short-chain fatty acids (SCFAs), secondary bile acids (BAs) and neurotransmitters (16). A complex two-way association network between the intrinsic gut microbiota and the brain (17), which is also known as the “microbial-gut-brain axis.” The microbial-gut-brain axis improves sleep disorders by affecting brain functions and behaviors (18–23).

Probiotics are defined as living microorganisms that have a health impact on the host, which have the following four properties (24): (1) easy to industrialized production; (2) survive from food production and storage; (3) tolerate the conditions of the hosts’ gastrointestinal tracts; (4) healthy for the host after consumption. Paraprobiotics are defined as nonviable microbial cells or crude cell extracts, which when administered in adequate amounts, confer a benefit on the consumer (25). Some lactic acid bacteria can alter the body’s stress sensitivity and response patterns to external stressful events, thereby improving sleep disorders (26, 27). For example, giving medical students who participated in autopsy courses a heat-inactivated, washed beverage containing cp2305 (a *Lactobacillus gasseri*) daily for 12 weeks can significantly improve sleep disorders (28). A study based on polysomnographic results in hypertensive individuals showed that compared with non-hypertensive individuals, hypertensive patients had increased apnea-hypopnea index (AHI), oxygen desaturation index (ODI), respiratory arousal index (RAI), and periodic limb movement index (PLMI). In addition, sleep efficiency (SE), the bruxism arousal index (BAI) and oxygen saturation (SpO2) level were decreased in the hypertensive group, showing significant obstructive sleep apnea (OSA) symptoms and suggesting a potential role for vitamin D in improving sleep arousals and sleep architecture (29). Meanwhile, vitamin D deficiency was found to be more prevalent in patients with OSA, which pointed to an independent association between vitamin D and OSA (30). Some clinical and experimental data suggested that probiotics may increase vitamin D levels. In a post-hoc analysis of a randomized controlled trial investigating the cholesterol-lowering efficacy of the bile salt hydrolase active *Lactobacillus reuteri* NCIMB 30242, it was surprising that the probiotic bacteria did not impaire the absorption of fat-soluble vitamins, and yet increased the mean circulating level of 25-Hydroxyvitamin D after 9 weeks of administration (31). So we hypothesized that probiotics could improve OSA, a chronic and sleep-related breathing disorder (30) by increasing vitamin D levels. With positive feedback from early experiments in animals (32), OSA has also been shown to improve directly in humans through restoring probiotics or gut microbiome homeostasis, as well as physiological barrier integrity via the gut-brain axis (33). In other clinical trials, probiotics are also shown to play a therapeutic role in the management of mood disorders, such as severe depression or anxiety, by modulating the “microbe-gut-brain axis.” Since sleep quality is a comprehensive reflection of physical and psychological conditions (34), and from the articles included in the meta-analysis, it can be found that the improvement of sleep disorders is closely related to emotional states, so we speculate that probiotics and paraprobiotics improve sleep disorders by improving stringent states and emotional states of depression and anxiety. Paraprobiotics are inactivated microbial cells or cellular components that can also improve sleep disorders (35). After taking administration of CP2305 orally in patients with irritable bowel syndrome (IBS), studies find that it can colonize the intestinal tract and continue to play a stress-relieving role with certain cellular components after inactivation (36, 37). Heat-inactivated paraprobiotic CP2305 shows a good effect on sleep states of medical students in stressful environments (38). A study (39) summarizing 36 articles associating gut microbiota found that regulating the abundance of key beneficial bacteria, such as CP2305, through a healthy diet can enhance metabolism, improve sleep, and support sleep homeostasis. Thus, we speculate that probiotics and paraprobiotics can be used as a popular clinical supplement to improve sleep disorders through the microbial-intestinal-brain axis.

Otherwise, a systematic review and meta-analysis (40) about effects of probiotics and paraprobiotics on subjective and objective sleep metrics show that probiotics or paraprobiotics supplementation may have some efficacy in improving perceived sleep health using the PSQI in sleep healthy adults and which with sleep disorders, while the evidence does not support a benefit of consuming probiotics or paraprobiotics when measured by other subjective or objective sleep scales.

Therefore, we included the references of high-quality randomized controlled trials and comprehensively and quantitatively evaluated the effects of probiotics and paraprobiotics on sleep disorders and sub-healthy sleep conditions with subjective and objective scales and subgroup classifications. The present study concluded that probiotics and paraprobiotics have a significant positive effect on the sleep quality of adults with sleep disorders or sub-healthy sleep conditions, which provides a meta-analysis evidence for the treatment of probiotics and paraprobiotics in sleep disorder and sub-healthy sleep conditions, as well as some suggestions on how to use potentially suitable probiotics.

## 2 Methods

### 2.1 Literature search strategy

We conducted a systematic literature search to identify studies on the effects of probiotics and paraprobiotics on sleep disorders. We searched four electronic databases (Pubmed, Embase, Cochrane Library and Web of Science) to find potentially pertinent articles up through November 2022 using Medical Subject Headings (MeSH) and free-text terms for “probiotic*” OR “probiotic agent” OR “paraprobiotic*” AND “sleep disorder*” OR “sleep disturbance*” OR “disorder*, sleep wake” OR “sleep wake disorder” OR “wake disorder*, sleep” OR “subwakefullness syndrome*” OR “syndrome* subwakefullness” OR “disorder*, sleep” OR “sleep-related neurogenic tachypnea*” OR “neurogenic tachypnea*, sleep-related” OR “tachypnea*, sleep-related neurogenic” OR “long sleeper syndrome*” OR “sleeper syndrome*, long” OR “syndrome*, long sleeper” OR “short sleeper syndrome*” OR “sleeper syndrome*, short” OR “syndrome*, short sleeper” OR “short sleeper phenotype*” OR “phenotype*, short sleep” OR “sleep phenotype*, short.” The language of the study was limited to English. Relevant articles were screened independently by four investigators (YB, WKY, WNR, and ZL) based on inclusion and exclusion criteria. When any discrepancies arose among the four researchers were resolved by negotiation. In addition, references of included articles were searched for additional studies to ensure that our search strategy identified all relevant studies. The therapeutic effects of probiotics on sleep problems will need to be demonstrated in more suitable clinical trials in the future (Table 1).

**Table 1:** Search strategies in PubMed, Embase, Cochrane Library and Web of Science.

Database	Search strategy
PubMed	(“probiotic*”[Mesh] OR “paraprobiotic*” OR “probiotic agent”) AND (“sleep disorder*”[Mesh] OR “sleep disturbance*” OR “disorder*, sleep wake” OR “sleep wake disorder” OR “wake disorder*, sleep” OR “subwakefullness syndrome*” OR “syndrome* subwakefullness” OR “disorder*, sleep” OR “sleep-related neurogenic tachypnea*” OR “neurogenic tachypnea*, sleep-related” OR “tachypnea*, sleep-related neurogenic” OR “long sleeper syndrome*” OR “sleeper syndrome*, long” OR “syndrome*, long sleeper” OR “short sleeper syndrome*” OR “sleeper syndrome*, short” OR “syndrome*, short sleeper” OR “short sleeper phenotype*” OR “phenotype*, short sleep” OR “sleep phenotype*, short”)
Embase	(“probiotic*”/exp. OR “paraprobiotic*” OR “probiotic agent”) AND (“sleep disorder*”/exp. OR “sleep disturbance*” OR “disorder*, sleep wake” OR “sleep wake disorder” OR “wake disorder*, sleep” OR “subwakefullness syndrome*” OR “syndrome* subwakefullness” OR “disorder*, sleep” OR “sleep-related neurogenic tachypnea*” OR “neurogenic tachypnea*, sleep-related” OR “tachypnea*, sleep-related neurogenic” OR “long sleeper syndrome*” OR “sleeper syndrome*, long” OR “syndrome*, long sleeper” OR “short sleeper syndrome*” OR “sleeper syndrome*, short” OR “syndrome*, short sleeper” OR “short sleeper phenotype*” OR “phenotype*, short sleep” OR “sleep phenotype*, short”)
Cochrane library	((probiotic*):ti,ab,kw OR (paraprobiotic*):ti,ab,kw OR (probiotic agent):ti,ab,kw) AND ((sleep disorder*):ti,ab,kw OR (sleep disturbance*):ti,ab,kw OR (disorder*, sleep wake):ti,ab,kw OR (sleep wake disorder):ti,ab,kw OR (wake disorder*, sleep):ti,ab,kw OR (subwakefullness syndrome*):ti,ab,kw OR (syndrome* subwakefullness):ti,ab,kw OR (disorder*, sleep):ti,ab,kw OR (sleep-related neurogenic tachypnea*):ti,ab,kw OR (neurogenic tachypnea*, sleep-related):ti,ab,kw OR (tachypnea*, sleep-related neurogenic):ti,ab,kw OR (long sleeper syndrome*):ti,ab,kw OR (sleeper syndrome*, long):ti,ab,kw OR (syndrome*, long sleeper):ti,ab,kw OR (short sleeper syndrome*):ti,ab,kw OR (sleeper syndrome*, short):ti,ab,kw OR (syndrome*, short sleeper):ti,ab,kw OR (short sleeper phenotype*):ti,ab,kw OR (phenotype*, short sleep):ti,ab,kw OR (sleep phenotype*, short):ti,ab,k)
Web of science	((((((((((((((((((((((TS = (probiotic*)) OR TS = (paraprobiotic*)) OR TS = (probiotic agent)) AND TS = (sleep disorder*)) OR TS = (sleep disturbance*)) OR TS = (disorder*, sleep wake)) OR TS = (sleep wake disorder)) OR TS = (wake disorder*, sleep)) OR TS = (subwakefullness syndrome*)) OR TS = (syndrome* subwakefullness)) OR TS = (disorder*, sleep)) OR TS = (sleep-related neurogenic tachypnea*)) OR TS = (neurogenic tachypnea*, sleep-related)) OR TS = (tachypnea*, sleep-related neurogenic)) OR TS = (long sleeper syndrome*)) OR TS = (sleeper syndrome*, long)) OR TS = (syndrome*, long sleeper)) OR TS = (short sleeper syndrome*)) OR TS = (sleeper syndrome*, short)) OR TS = (syndrome*, short sleeper)) OR TS = (short sleeper phenotype*)) OR TS = (phenotype*, short sleep)) OR TS = (sleep phenotype*, short)

### 2.2 Inclusion and exclusion criteria

In accordance with PRISMA recommendations (41), all studies included in the initial search rigorously met the criteria of the Population, Intervention, Comparison, Outcome, and Study Design (PICOS) framework.

Included studies met the following criteria: (1) Patients aged ≥18 years who met any of the recognized diagnostic criteria for sleep disorders or healthy individuals with sleep disorder problems by psychological problems such as stress or emotions. (2) Trials including probiotic or paraprobiotic interventions (any dose, frequency, duration of intervention, route of administration, or strain type) that influence sleep quality. (3) The control group was set to the same conditions, except that it had no probiotic or paraprobiotic intervention. (4) At least one sleep quality outcome was measured. (5) The study design was randomized controlled trial (RCT).

Excluded studies met the following criteria: (1) Article title and abstract were not relevant to the topic of this study. (2) Duplicate literature. (3) Non-randomized controlled trials. (4) Non-clinical trials mainly included original research papers on animals, review, clinical case report and conference proceeding. (5) Studies without experimental data (including studies with non-normally distributed data). (6) Non-English literature. (7) Experimental subjects were adults and minors who were healthy and did not have sleep disorder problems (for whom no sleep disorder was modeled). (8) Studies focusing on patients with diseases indirectly impacting sleep quality, and the results about sleep are based on the impact of the primary disease.

### 2.3 Risk-of-bias and quality assessment

Two authors (WKY and WNR) independently assessed the risk of bias using the Cochrane Collaboration tool (42), judging the risk as “low,” “unclear” or “high” in the following aspects: selection bias (including random sequence generation and allocation concealment), performance bias, attrition bias, detection bias, reporting bias and other biases. Regarding other biases, potential bias such as “only men” related to the specific study was evaluated as “high risk”; No written reference as “unknown risks”; It was evaluated as “low risk” only when the article clearly stated that there was no obvious other bias. When there was a disagreement, a third author (YB) became involved, and a consensus was reached.

### 2.4 Data synthesis and analysis

Two authors (YB and WNR) read each study independently and extracted relevant data according to the Cochrane Handbook for Systematic Reviews of Interventions, “Checklist of Items to Consider in Data Collection or Data Extraction” (43), including the following detailed information: authors, year of publication, country, demographic characteristics (e.g., age, sex, sample size, and sample type), probiotic characteristics (e.g., species and intervention duration), and sleep-related outcomes (all continuous variable data).

In the studies, we used a random-effects model to measure the effects of probiotics intervention between the intervention and control groups by the standardized mean difference (SMD) of the mean change from baseline. Consistent with convention, the SMD was assessed using Cohen’ g as a measure of effect size; 0.2–0.5 was interpreted as small, 0.5–0.8 as medium, and ≥ 0.8 as large.

Regarding the extraction of sleep-related outcome data: (1) We directly extracted the mean and standard deviation of the pre-*vs* post-treatment change between the intervention and control groups of the experiment on probiotic or parabiotic interventions for sleep disorders, and then applied statistical methods to the pre-*vs* post-treatment data of the two groups (MEAN ± SD) to compute the difference and the corresponding formula (42). (2) The difference data between the intervention and control groups were entered into the STATAMP16 (64bit) version of the software to calculate the difference in treatment effect between the groups, which was reflected by the *p*-value (a *p*-value of < 0.05 was considered statistically significant).

When the article was without specific data or only with figures and graphs available, we requested unpublished data from the authors. If we were unable to obtain these data, we used GetData Graph Digitizer software to estimate the data from the figures or graphs. Raw data were estimated based on the axes and the mean and SD were recorded by punctuation. If direct SD data could not be extracted, the SE was converted to SD based on the guidelines (42), where n is the sample size. If direct data or graphs labeled ΔSD were available then that data was extracted directly.

### 2.5 Subgroup analysis

Subgroup analysis was conducted to assess whether the manifestation of the improvement effect of probiotics or paraprobiotics on sleep disorders would be affected by the subjectivity and objectivity of the final data. Sleep latency is important objective sleep characteristic (44). There are many types of subjective indicators and it is impossible to generalize the unitive data. Our study used the Pittsburgh sleep quality index (PSQI) as the primary indicator, so after doing the heterogeneity analysis of the subjective and objective indicators, we continued to do the subgroup analysis about the outcome reflected by the type of subjective indicator. Since there are fewer indicators related to the effect of probiotics or paraprobiotics on the improvement of sleep disorders by gender, it is impossible to carry out subgroup analysis, so we will discuss the available data and refer to the discussion section later.

**Figure 1:**
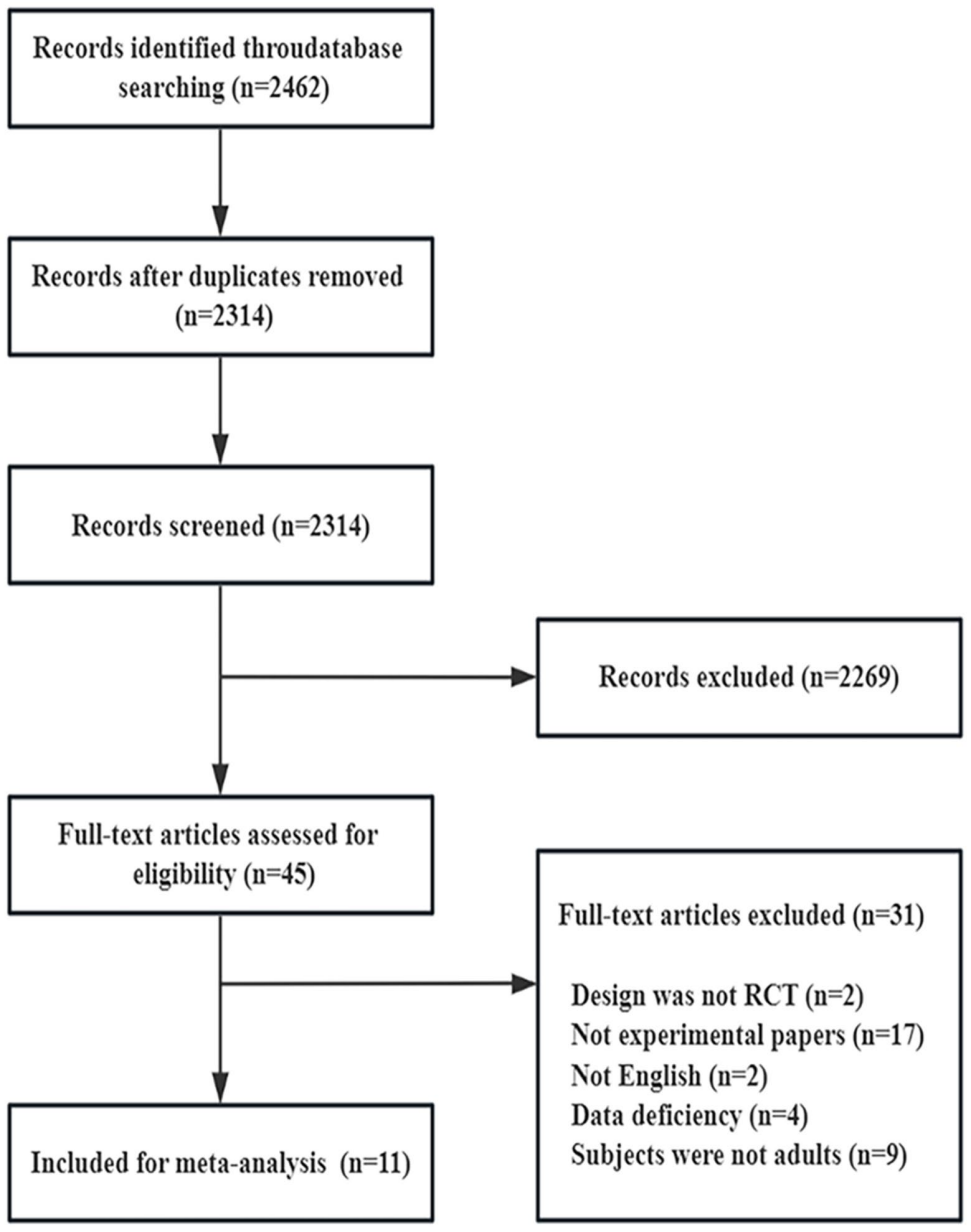
PRISMA flow diagram illustrating the study selection process. PRISMA, preferred reporting items for systematic reviews and meta-analyses.

In addition, Subgroup analysis was conducted to assess whether effect size was affected by country, probiotic species, duration of regimen, etc., and to assess heterogeneity. We tested between studies heterogeneity using Cochran’s Q test and *I*^2^ statistics. *I*^2^ values of 25–50%, 50–75%, and ≥75%, respectively, indicated low, moderate, and high heterogeneity. A *p*-value of <0.10 for Cochran’s Q was used to indicate significant heterogeneity (45). To find possible sources of heterogeneity, we performed sensitivity analysis, using STATAMP version 16.0 (64bit) software, and the data were described as effect size ±95% confidence interval (CI). Finally, funnel plots were used to exclude and examine the possibilities of possessing reporting bias in the studies (Figure 1).

## 3 Results

### 3.1 Study inclusion

The included articles are presented in Table 2. A total of 11 articles were included, categorized as 7 articles related to artificial sleep disorders models (such as students preparing for exams) or those in a sub-healthy sleep state, 3 articles related to sleep disorders caused by emotional disorders such as depression, anxiety, or stress, and 1 article specifically reporting on insomnia patients. Out of the eleven studies, six studies measured brainwave parameters as sleep latency index here, and nine studies provided subjective sleep scale such as Pittsburgh sleep quality index (PSQI), Insomnia Severity Index (ISI), Athens insomnia scale (AIS), Epworth Sleepiness Scale (ESS), and obstructive sleep apnea (OSA) data. When a study has different types of data, we tend to choose the same type of data as other studies. Table 2 describes the general characteristics of these 11 studies. The age data is presented as MEAN ± SD.

**Table 2:** Characteristics of included studies.

Author(s)	Country	Sample size (EG/CG)	Age (mean; years)	Probiotic	Duration	Population characteristics	Type	Contrast
Nishida et al. (38)	Japan	16/16	21.03 ± 2.80	*Lactobacillus gasseri* CP2305	5 weeks	Medical students (sleep disorder model)	RCT	Placebo beverage prepared from lactic acid-acidified, non-fermented milk, without heat-inactivated CP2305 (paraprobiotic CP2305)
Nishida et al. (28)	Japan	34/35	25.00 ± 2.12	*Lactobacillus gasseri* CP2305	12 weeks	Medical students (sleep disorder model)	RCT	Placebo beverage without CP2305 powder
Takada et al. (59)	Japan	48/46	22.70 ± 1.34	*Lactobacillus casei* strain Shirota (LcS)	11 weeks	Medical students (sleep disorder model)	RCT	Placebo milk without LcS
Sawada et al. (60)	Japan	12/12	24.00 ± 0.00	*Lactobacillus gasseri* CP2305	11 weeks	Medical students (sleep disorder model)	RCT	Placebo beverage without CP2305 powder
Lee et al. (61)	Switzerland	63/59	38.26 ± 10.94	*Lactobacillus reuteri* NK33 + *Bifidobacterium adolescentis* NK98 (NVP-1704)	8 weeks	Healthy adults with psychological stress and subclinical symptoms of depression or anxiety	RCT	Placebo capsule, made of maltodextrin and without probiotic NVP-1704
Ho et al. (62)	Switzerland	12/9	27.10 ± 20.19	*Lactobacillus plantarum* PS128 (PS128)	30 days	Insomniacs	RCT	Placebo capsules with only microcrystalline cellulose
Nishida et al. (63)	Japan	31/29	25.09 ± 3.04	*Lactobacillus gasseri* CP2305	24 weeks	Medical students (sleep disorder model)	RCT	Placebo tablets without the lactic acid bacteria powder
Moloney et al. (64)	Iran	20/20	20.7 ± 1.25	*Bifidobacterium longum* 1714	8 weeks	Volunteers (sleep disorder model)	RCT	Placebo capsules without *B. longum* AH1714
Baião et al. (65)	UK	24/25	28.8 ± 8.93	Consisted of 14 species of bacteria	4 weeks	Volunteers with moderate depression	RCT	Placebo capsules without live bacteria
Zhu et al. (66)	China	30/30	22.4 ± 0.27	*Lactobacillus plantarum* JYLP-326	3 weeks	Medical students (sleep disorder model)	RCT	Placebo powder with maltodextrin
Patterson et al. (56)	Germany	44/47	23.48 ± 4.30	Lpc-37	5 weeks	Volunteers with high chronic stress	RCT	Placebo capsules without Lpc-37

EG, experimental group; CG, control group.

### 3.2 Study quality

**Figure 2:**
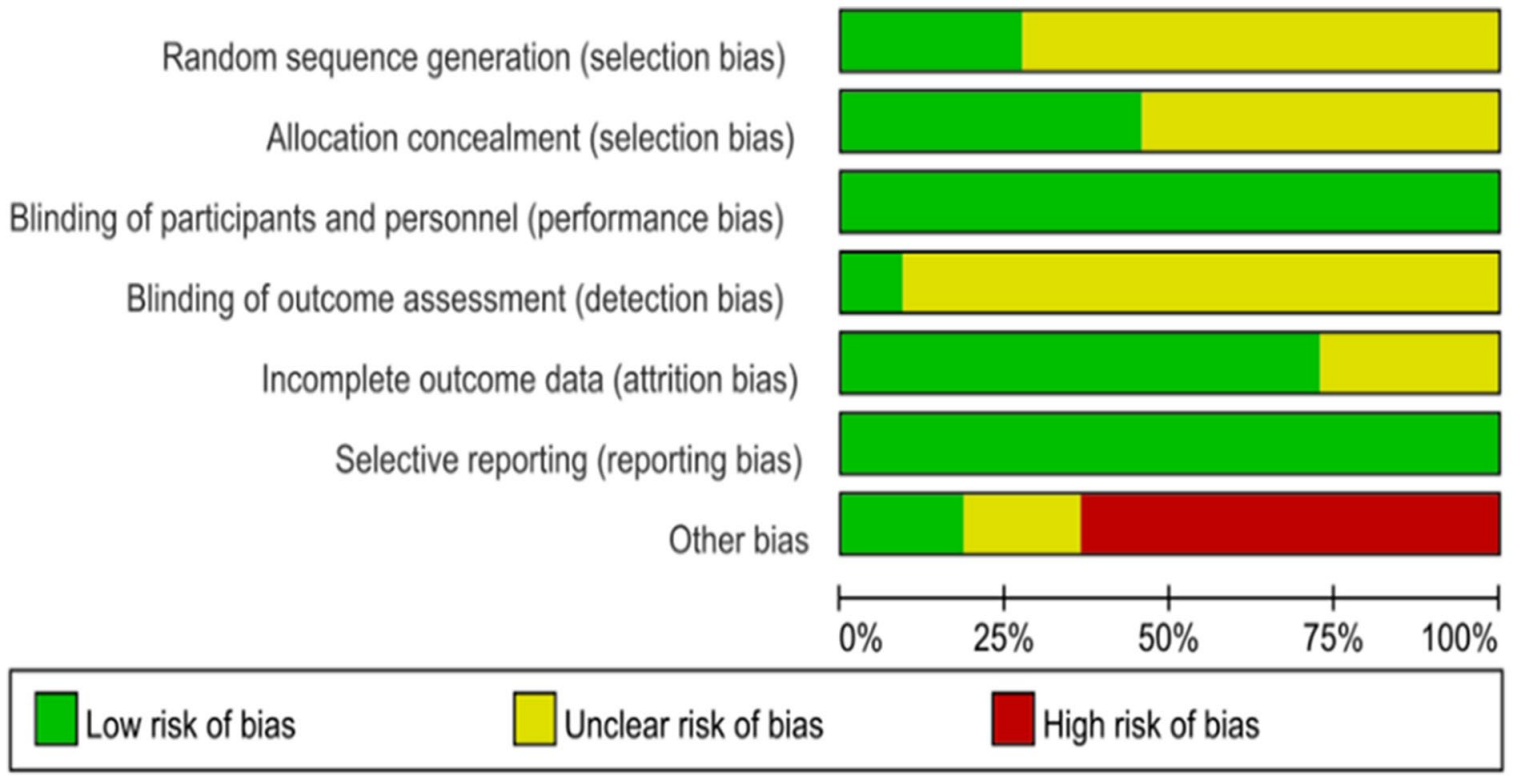
Risk-of-bias assessments of the included studies (domains from the Cochrane Handbook for Systematic Reviews of Interventions).

**Figure 3:**
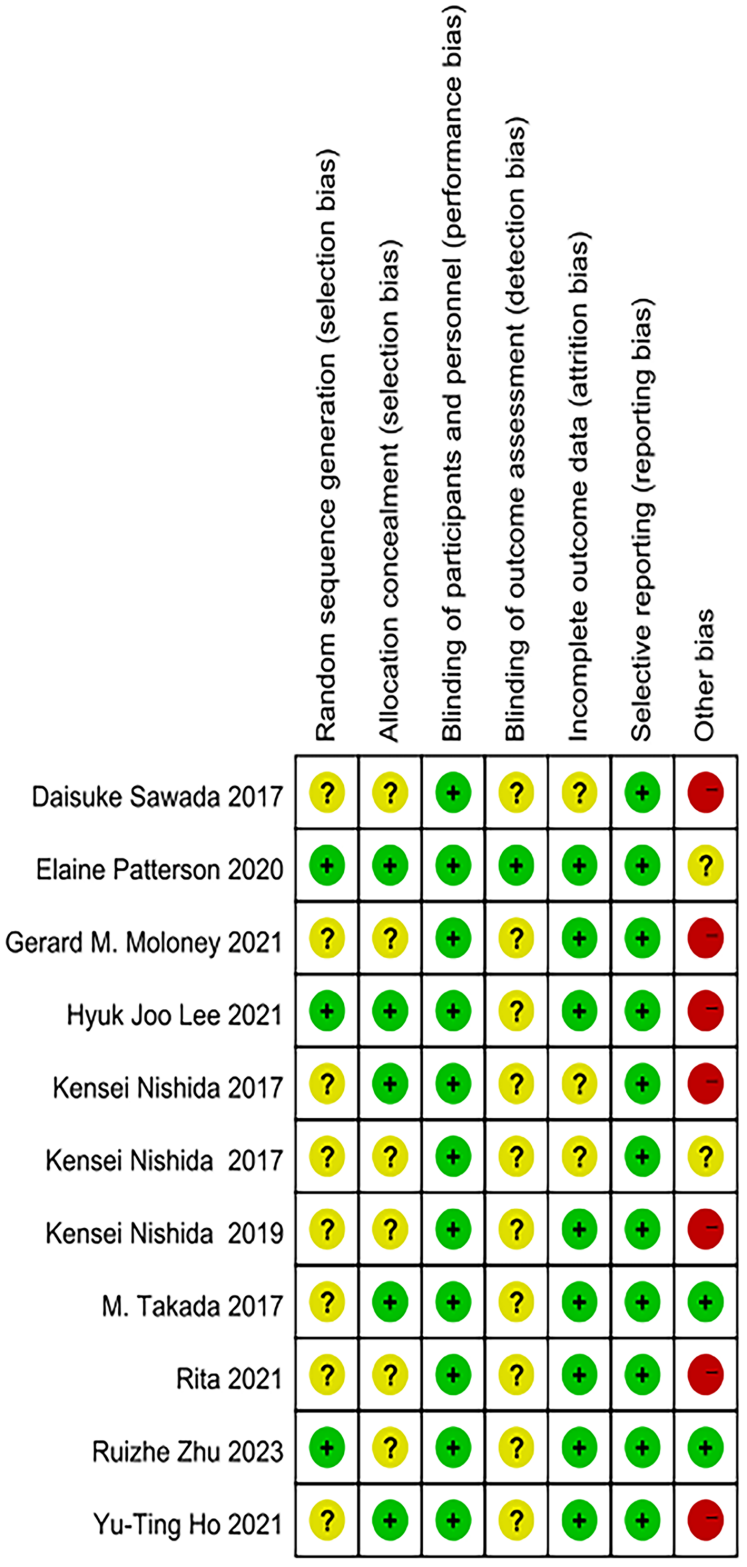
Risk-of-bias assessment of each study (domains from the Cochrane Handbook for Systematic Reviews of Interventions).

The results of the quality assessment of the included studies are shown in Figures 2, 3. All 11 studies were randomized, but most (*n* = 8) did not describe the method of randomization. Most (*n* = 6) did not sufficiently describe the method of concealment, but all (*n* = 10) were double-blinded (researchers and participants). This suggested a “high” risk of selection bias and a “low” risk of performance bias. Most studies did not agree to their study, with only a few (*n* = 1) explicitly mentioning that the experimental design was unlikely to break blinding. In terms of risk assessment for attrition bias, it was determined to be “low” if there were no participant dropouts or if there were dropouts but explicit reasons were provided, the article stated that missing data did not affect the outcomes, and the article mentioned remedial measures. It was determined to be “high” if the article explicitly stated that missing data had an impact on the outcomes. If there was insufficient information to determine these two levels, it was classified as “unclear” (*n* = 3). All predetermined outcomes of all articles were reported (*n* = 11). Regarding other biases, this article determined that only when it explicitly stated or was comprehensively considered that the full text was unbiased that there were no obvious other biases could it be considered “low” (*n* = 2). If the article had potential biases related to specific research designs (such as only including males), it was considered “high” (*n* = 7). In all other cases, it was deemed “unclear” (*n* = 2).

### 3.3 Main efficacy of meta-analysis

#### 3.3.1 Efficacy of probiotics and paraprobiotics in the treatment of sleep disorders

**Figure 4:**
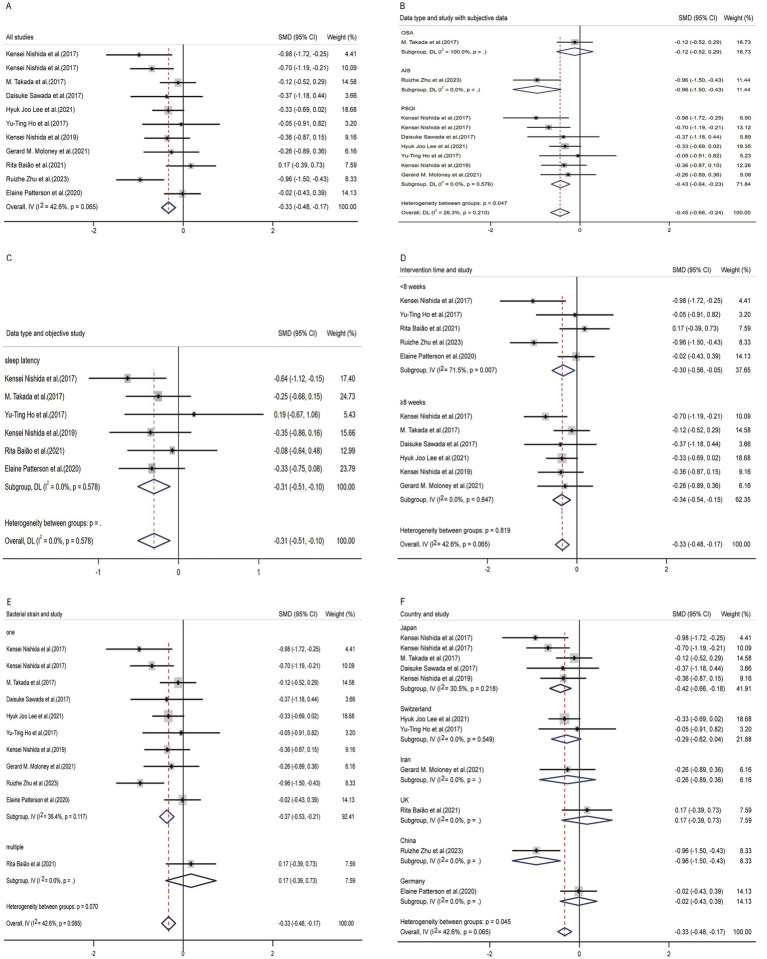
Forest plot of random-effects model meta-analysis of the effect of probiotics on sleep disturbance. **(A)** The overall effect of probiotics on sleep disturbance. **(B)** Subgroup analysis of subjective indicators. **(C)** Subgroup analysis of objective indicators. **(D)** Subgroup analysis according to duration of intervention. **(E)** Subgroup analysis according to strains of probiotics supplement. **(F)** Subgroup analysis according to different countries. CI, confidence interval; P, heterogeneity *p*-value; SMD, standardized mean difference; AIS, Athens insomnia scale; OSA, obstructive sleep apnea; PSQI, Pittsburgh sleep quality index (the study used a random-effects model).

In the process of analyzing the 11 articles involved in our study, a random effects model was used (overall effect size: −0.34 [−0.56 to −0.13]; *I*^2^ = 42.6%; *p* = 0.001; Figure 4A). The overall data indicated that probiotics or paraprobiotics supplements had a positive therapeutic effect on treating sleep disorders and sleep sub-healthy states.

#### 3.3.2 Subgroup analysis of subjective indicators

In nine studies providing pre-and post-treatment subjective sleep data, three studies presented multiple data types, including Epworth Sleepiness Scale (ESS), Insomnia Severity Index (ISI) and Pittsburgh sleep quality index (PSQI). In order to reduce the impact of different data types on the final result, we only selected PSQI as the indicator for articles that provide multiple data types. From the perspective of subjective questionnaires, the improvement of probiotics and paraprobiotics on sleep disorders was evident (Figure 4B). After conducting subgroup analysis, this study found significant heterogeneity (*p* = 0.000), indicating that there was a significant difference between the experimental group and the control group. That is to say, probiotics or paraprobiotics have significant therapeutic significance for sleep disorders from the perspective of subjective sleep indicators. We also conducted a subgroup analysis of subjective sleep quality indicators to test whether the heterogeneity among the three types of subjective sleep scales was due to inconsistent data types. Cochran’s Q statistics for heterogeneity showed that *p* = 0.210, indicating that this grouping factor did not affect the positive outcome reflected by the overall subjective sleep scale.

#### 3.3.3 Subgroup analysis of objective indicators

In Figure 4C, it could be seen that 6 studies provided objective sleep electroencephalographic data (sleep latency), and it could also be seen that probiotics and paraprobiotics had a positive effect on improving sleep quality indicators (*p* = 0.003).

#### 3.3.4 Subgroup analysis according to duration of intervention

In Figure 4D, we categorized different intervention durations and compared the efficacy between the two groups using an 8-week cutoff. The results showed that the group with a longer intervention duration had better effects compared to the group with a shorter intervention duration.

#### 3.3.5 Subgroup analysis according to strains of probiotics supplement

Figure 4E presented an analysis based on the number of species contained in probiotics and paraprobiotics. Due to limitations in the integrity of the data in the article, the efficacy of different subgroups of probiotics and paraprobiotics was analyzed against the background of sleep latency data. Although the small effect size showed a small *p*-value (*p* = 0.003), there was only one study that used two or more strains of probiotics, so the result of this analysis (i.e., the efficacy of a single strain of probiotic substance is superior to the simultaneous use of multiple strains) could not be considered a general conclusion.

#### 3.3.6 Subgroup analysis according to different countries

Figure 4F displayed the results grouped by different countries. Although the combined effect size was *p* = 0.001, indicating a minimal influence of country on the outcome measure, it was not recommended to directly interpret this analysis as a general conclusion due to the limited number of studies from each country.

The participants included in this study were adults who already had sleep disorders or were in a sub-healthy sleep state. From the data, it could be observed that probiotics and paraprobiotics had a more significant therapeutic effect (sleep disorder model) (*p* = 0.000) on initially healthy adults. However, for individuals with sleep disorders caused by emotional issues (stress, depression, anxiety) or insomnia existed before, the treatment was effective but not statistically significant (*p* = 0.404).

#### 3.3.7 Heterogeneity, sensitivity and bias analysis

**Figure 5:**
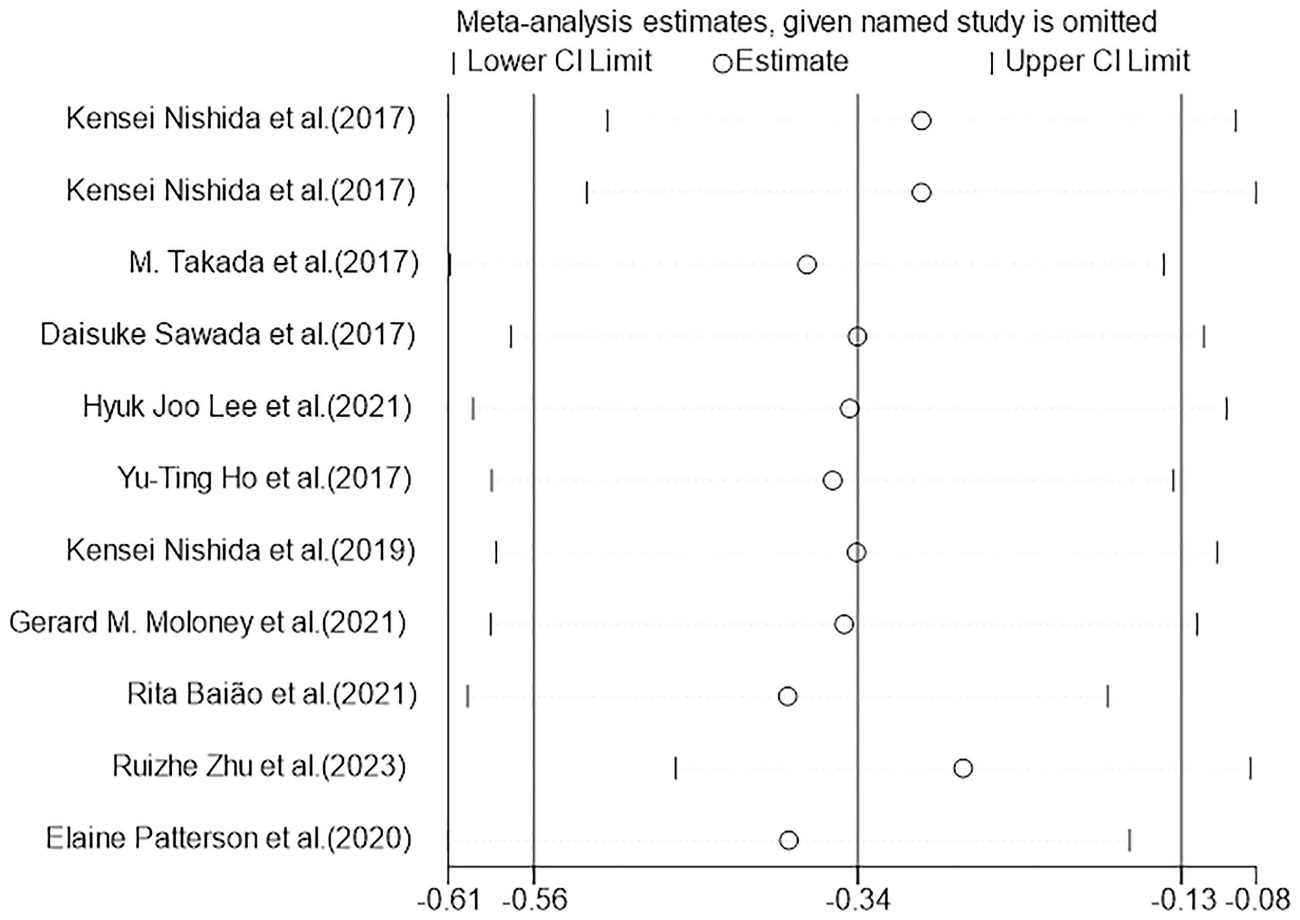
Heterogeneous graph of random-effects model meta-analysis of the overall effect of probiotics on cognition. CI, confidence interval (the study used a random-effects model).

**Figure 6:**
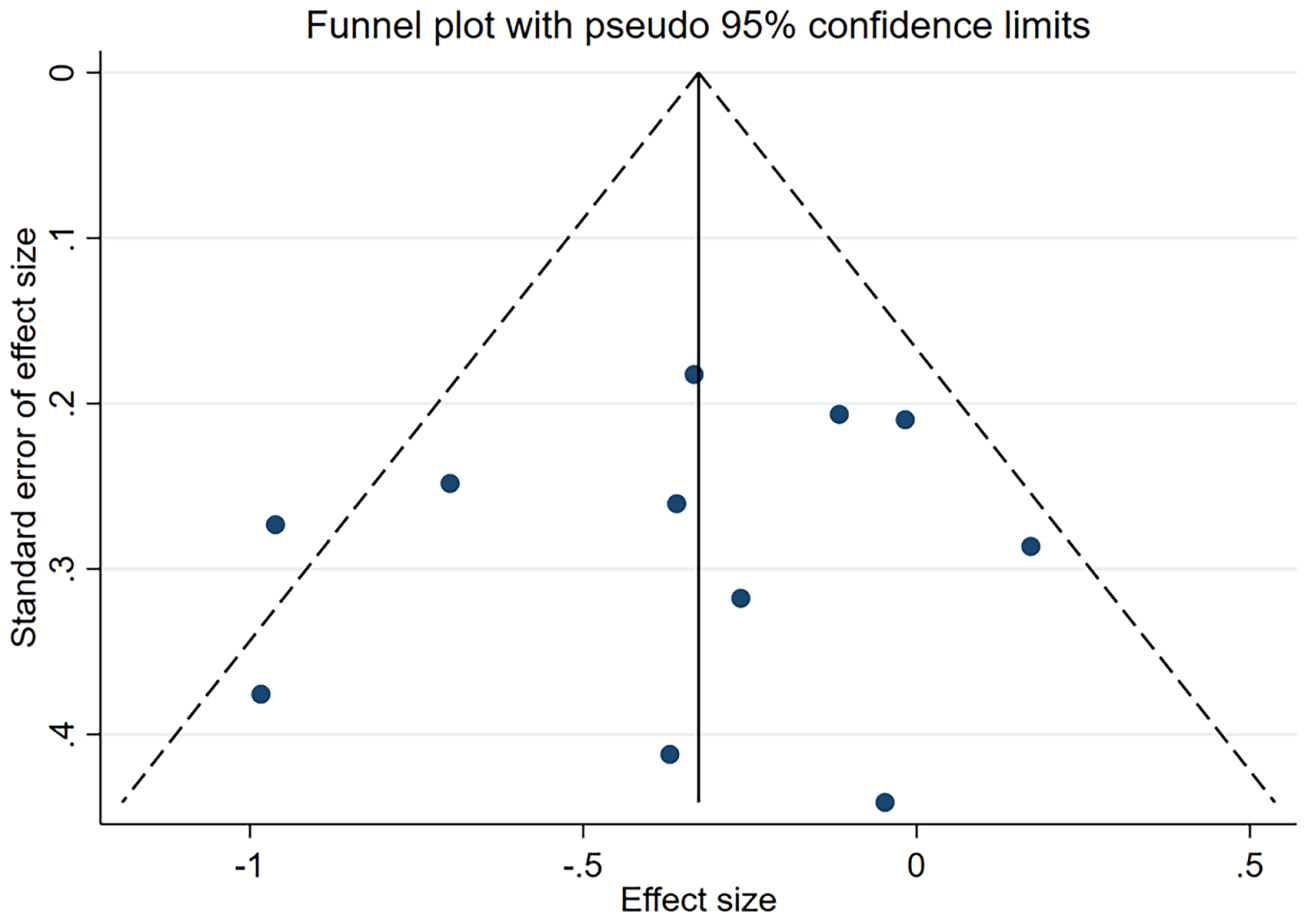
Sensitivity analysis of random-effects model meta-analysis of the overall effect of probiotics on cognition. CI: confidence interval (the study used a random-effects model).

Figures 5, 6 demonstrated that a small number of studies (n = 4) showed a significant impact of certain variables on the results, indicating insufficient robustness. However, most of studies (*n* = 7) had trustworthy results. Additionally, the points on the funnel plot were evenly distributed on both sides of the vertical line, suggesting no publication bias despite the relatively small sample size. Overall, this study had low heterogeneity.

## 4 Discussion

All the studies included were randomized controlled trials (RCTs), which further increased their rigor and credibility. The overall results of our meta-analysis indicated that the intake of probiotics and paraprobiotics could significantly improve the level of sleep disorders and sleep sub-health states, wherein the subjective sleep index had significant positive effect. Furthermore, though the subjects included were in a wide range, involving sleep disorders model and patients with sleep sub-health states, the results still showed positively. So, we inferred those adults with sleep disorders, including young person not included in the study and healthy sleepers, could improve or prevent decreased sleep quality by supplementing with probiotics and paraprobiotics. As a strain that is generally considered to be safe for humans (46), the intake of probiotics will be an effective way to improve sleep disorders.

Although there is insufficient evidence, that the supplementation of probiotics and paraprobiotics was an effective way to improve sleep disorders, the functional connection between gut microbes and the brain had been well established. Gut microbiota profoundly regulate communication between gut and brain (27) through neural, endocrine, and immune signaling pathways (26, 47) under the microbiota-gut-brain axis (48). For example, in stress states, the brain senses psychological stress and triggers the release of corticotropin-releasing factor (CRF) from the paraventricular nucleus of the hypothalamus, which activates the hypothalamic–pituitary–adrenal axis (HPA) and sympathetic nervous system (SNS), and the stress response triggers by CRF can lead to decreased sleep quality (49, 50). Meanwhile, probiotics interact with the nervous and endocrine systems, which reduces the response of HPA to stress and improves sleep disorders (51–53). The subjective indexes in our study included OAS, AIS, and PSQI. Due to the small overall heterogeneity of the subjective index, the result of the subjective sleep scales could show a significant improvement in sleep disorders. And subjective indexes, by contrast, were more significant than objective indexes in terms of improvement in sleep disorders. At the same time, the results of some studies (40) showed that the overall effect size of objective electroencephalogram sleep parameters (including sleep latency) were small and positive, but not significant, this was also similar to our findings. Of the six objective sleep indexes included in this study, one index was sleeping latency in the first N3 stage (N3, also known as slow-wave sleep, represents the deepest sleep stage of non-rapid eye movement sleep.), which was also included to maintain data integrity. Subjective good sleep has been reported to be associated with sleep efficiency and the amount of slow-wave sleep (SWS) (54), while a systematic review (55) has reported that SWS decrease with increasing daily life stressors and experimental psychological stressors. Therefore, the inclusion of this slow wave sleep data is also beneficial to ensure the validity of the results of objective sleep quality indexes improvement.

In addition, in terms of duration (8 weeks as the boundary), the subgroup results showed that the beneficial effects on sleep disorders were more obvious when the intervention duration was greater than or equal to 8 weeks. However, there is controversy regarding the duration of intervention as a variable in measuring the effects of probiotics on sleep disorders. A research (16) has pointed out that there is no significant difference among trials lasting 8 weeks or more relative to those with shorter durations. This might be because our study included patients with health conditions such as depression and anxiety, whereas another study focused solely on healthy subjects. Perhaps for individuals with sleep disorders, the treatment might be more effective due to a larger opportunity window. Additionally, subjects with different health conditions have different gut microbiota compositions, indicating that the same treatment may elicit different responses.

Results of some research showed that in terms of the sleep-improving effect of probiotics and paraprobiotics, PSQI scores of males were improved, but not that of females (*p* = 0.760) (38). Compared with males, females had lower sleep-related recovery scores (56). Another study (28) mentioned that females had a significant improvement in sleep latency indexes after 12 weeks of intervention with the paraprobiotic CP2305, but this significance might be due to that females had higher initial anxiety scores than males. There is also study (16) emphasizing that the effects of probiotics are sex dependent, and the conclusions and inferences align with ours. Therefore, we tend to favor probiotics and paraprobiotics for better therapeutic effects in male patients with sleep disorders. Due to the insufficient number of subjects, we hope to explore more literature and more generalizations in the near future.

The main strength of this meta-analysis was that we obtained data of sleep improvement from all relevant clinical randomized controlled studies and derived quantitative results. To our knowledge, there are currently few studies about quantifying the effects of probiotics and paraprobiotics on improving sleep sub-health states in patients with insomnia and in artificial sleep disorder models. Our study focused on adults and included both subjective and objective sleep scales as well as related subgroup analyses. Although it has proved that probiotic supplements significantly reduce PSQI scores, this is limited to subjective measures (57). Furthermore, the subjects included in this study also comprised other conditions that indirectly affected sleep quality, such as fibromyalgia, cirrhosis, and irritable bowel syndrome (IBS). The indicators of changes in sleep quality are presented as secondary indicators related to the improvement of the primary disease. Therefore, we have reason to suspect that the improvement in sleep quality observed in these studies due to probiotic supplementation may actually be achieved through the amelioration of the primary diseases, and thus cannot be directly cited as evidence for probiotics improving sleep disorders. In addition, the meta-analysis provided supplementary recommendations for some detailed probiotic substances: (1) Probiotic substances can be used in people with sub-healthy sleep as a dietary supplement to improve or prevent sleep disorders; (2) The duration of interventions can be appropriately prolonged; (3) By supplementing prebiotics, engineered bacteria, or adopting fecal microbiota transplantation (FMT) method to balance the microenvironment of the gut microbiota and promote probiotics’ growth (58). Nevertheless, we had to acknowledge several limitations of the study. First, we inevitably used some digital software to obtain some image-only results, which meant that there might be a certain amount of error. Second, the sample sizes of our included studies were generally small, which would also lead to large standard errors of the end results. Third, strains, doses, and durations varied among studies, and the conclusion was not generalizable. Fourth, the samples we included were mainly from develop countries. Fifth, assessment of sleep disorder lacked consistency and objectivity, which needed more accurate evaluation techniques. Therefore, more high-quality studies are needed to verify the conclusions of this paper.

## 5 Conclusion

Our meta-analysis demonstrated that the consumption of probiotics and paraprobiotics had a significant positive effect on the sleep quality of adults with sleep disorders or sub-healthy sleep conditions. Probiotic therapy is an effective and accessible method that can be incorporated into daily life as a dietary supplement to improve and prevent symptoms of sleep disorders. Subgroup analysis revealed that probiotics and paraprobiotics showed a more significant improvement in subjective data compared to objective data. Considering the individual differences in both strains and human bodies, we suggest customizing different approaches for different individuals to maximize efficacy. Additionally, we also suggest longer intervention periods for individuals with sleep disorders. Further high-quality researches are needed to understand the detailed mechanisms of various probiotics and paraprobiotics and to recommend specific formulations.

## Data Availability

The original contributions presented in the study are included in the article/supplementary material, further inquiries can be directed to the corresponding author.
